# Identification of two novel mutations in POU4F3 gene associated with autosomal dominant hearing loss in Chinese families

**DOI:** 10.1111/jcmm.15359

**Published:** 2020-05-11

**Authors:** Xiaohui Bai, Fengguo Zhang, Yun Xiao, Yu Jin, Qingyin Zheng, Haibo Wang, Lei Xu

**Affiliations:** ^1^ Otologic Center Shandong Provincial ENT Hospital, Cheeloo College of Medicine Shandong University Jinan China; ^2^ Department of Clinical Laboratory Shandong Provincial Hospital, Cheeloo College of Medicine Shandong University Jinan China; ^3^ Department of Otolaryngology‐Head & Neck Surgery Case Western Reserve University Cleveland OH USA

**Keywords:** autosomal dominant, Chinese family, hearing loss, identification, novel mutation, POU4F3

## Abstract

Autosomal dominant non‐syndromic hearing loss is genetically heterogeneous with 47 genes identified to date, including *POU4F3*. In this study, by using a next‐generation sequencing panel targeting 127 deafness genes, we identified a pathogenic frameshift mutation c.704_705del and a missense mutation c.593G>A in two three‐generation Chinese families with late‐onset progressive ADNSHL, respectively. The novel mutations of *POU4F3* co‐segregated with the deafness phenotype in these two families. c.704_705del caused a frameshift p.T235fs and c.593G>A caused an amino acid substitution of p.R198H. Both mutations led to an abnormal and incomplete protein structure. *POU4F3* with either of the two mutations was transiently transfected into HEI‐OC1 and HEK 293 cell lines and immunofluorescence assay was performed to investigate the subcellular localization of mutated protein. The results indicated that both c.704_705del (p.T235fs) and c.593G>A (p.R198H) could impair the nuclear localization function of POU4F3. The p.R198H POU4F3 protein was detected as a weak band of the correct molecular weight, indicating that the stability of p.R198H POU4F3 differed from that of the wild‐type protein. While, the p.T235fs POU4F3 protein was expressed with a smaller molecular weight, implying this mutation result in a frameshift and premature termination of the POU4F3 protein. In summary, we report two novel mutations of *POU4F3* associated with progressive ADNSHL and explored their effects on POU4F3 nuclear localization. These findings expanded the mutation spectrum of *POU4F3* and provided new knowledge for the pathogenesis of *POU4F3* in hearing loss.

## 1 INTRODUCTION

Hearing loss is the most frequent sensory impairment in humans, with a morbidity of 1/1000 in newborns.^1^ Based on available evidence, approximately 60% of all cases are because of genetic factors. According to clinical characteristics, hearing loss can be divided into syndrome hearing loss and non‐syndrome hearing loss (NSHL). Non‐syndrome hearing loss accounts for about 70% of all hereditary cases.^2^ The hereditary modes of NSHL include autosomal recessive, autosomal dominant and X chromosome‐linked or mitochondrial inheritance, the incidences of which are, respectively, 80%, 20% and <1%.^3^ For autosomal dominant non‐syndrome hearing loss (ADNSHL), only 72 loci and 47 corresponding genes have been identified as causative factors (http://hereditaryhearingloss.org/). The genetic aetiology of most patients with ADNSHL cannot be diagnosed. Furthermore, because of the high genetic heterogeneity of NSHL, using conventional Sanger sequencing to identify variants causing ARNSHL is extremely time‐consuming and expensive. In contrast, next‐generation sequencing (NGS), characterized by high throughput and low cost, has made it possible to identify nearly all mutations and genes involved in the onset and development of hearing loss.

*POU4F3*, characterized by two DNA‐binding POU domains, was expressed exclusively in cochlear, vestibular hair cells and sensory.^4^, ^5^ Function studies have demonstrated that *POU4F3* was required for the differentiation and maturation of hair cells and establishment of ear neural network, but not in fate determination of inner ear hair cells.^6^ For example, hair cells experienced a rapid, progressive apoptosis in *POU4F3* knockout mice during the late gestation and early postnatal period, which further reduced innervation and loss of sensory neurons and caused severe hearing loss and balance impairment.^7^ Other studies show that *POU4F3* is involved in the regulation of downstream targets, such as BDNF, NT‐3, Lhx3, Gfi1 et al, which play important roles in inner ear development.^8^, ^9^, ^10^, ^11^ *POU4F3* might be a candidate for hereditary hearing impairment because of its important role in hair cells.

Clinicians have made great efforts for the identification of pathogenic mutations for hearing loss. *POU4F3* is the earliest discovered deafness‐related gene to cause ADNSHL DFNA15. In 1998, Vahava et al first identified an 8‐base pair deletion in *POU4F3* leading to progressive hearing loss.^12^ Until now, more than 30 mutations of *POU4F3* were found to be associated with the pathogenesis of DNFA15, most of which occurred in DNA‐binding domains. According to previous studies, *POU4F3*‐related DFNA15 manifested as late‐onset bilateral, progressive hearing loss with down‐sloping audiometric configurations.^13^, ^14^, ^15^ For example, in a five‐generation Dutch family linked with p.L289F on *POU4F3*, the progression rate for DNFA15 was 0.8‐1.4 dB/year.^16^

Here, we performed mutation analysis on two Chinese families with ADNSHL by using NGS and Sanger sequencing. A missense mutation c.593G>A (p.R198H) and a frameshift mutation c.704_705del (p.T235fs) were identified as the causative factor for DNFA15 with a late‐onset progressive symptom. Furthermore, we also investigated the effects of these two mutations on the microscopic structure and subcellular location of POU4F3 in cells.

## 2 MATERIAL AND METHODS

### 2.1 Clinical features

**FIGURE 1:**
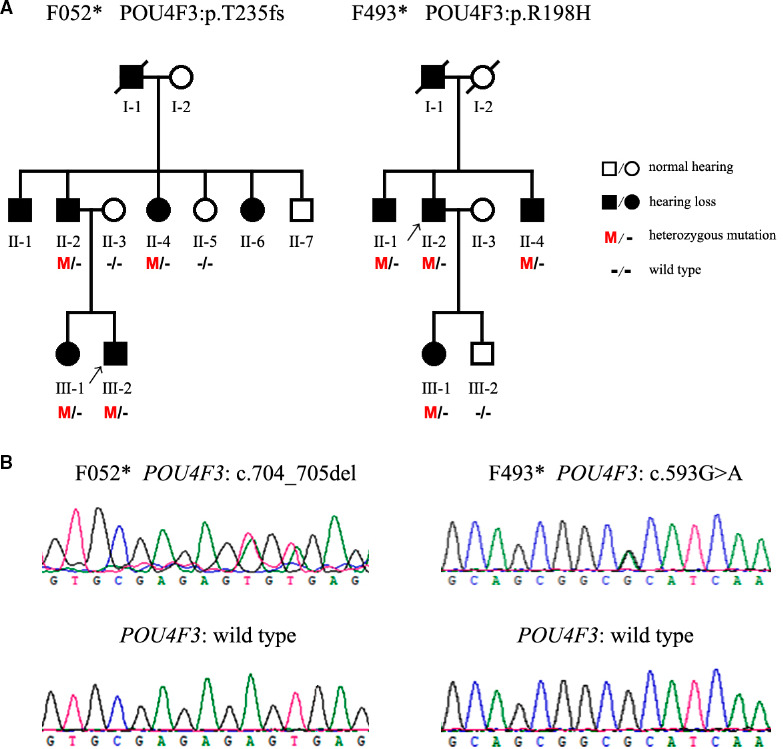
POU4F3 mutations identified in the two Chinese families suffering from autosomal dominant hearing loss. A, Pedigrees of the Chinese families. Black squares and circles represent members with symptoms of DFNA15. The proband is indicated by an arrow. M and—indicate the mutant and wild‐type alleles, respectively. Asterisks indicate the families with POU4F3 mutations identified in the present study. B, Sanger sequencing showing the c.704_705del (p.T235fs) and the c.593G>A (p.R198H) mutations in POU4F3

Two three‐generation Chinese families, respectively, named as F052* and F493*, have been recruited through Shandong Provincial ENT Hospital Affiliated to Shandong University. The pedigrees of the families with autosomal dominant pattern of inheritance are shown in Figure 1A. Due to patients' privacy or other reasons, there were only six members (II‐2, II‐4, III‐1, III‐2 with hearing loss and II‐3, II‐5 with normal hearing) in F052* and 5 members (II‐1, II‐2, II‐4, III‐1 with hearing loss and III‐2 with normal hearing) in F493* participating in our study (Figure 1).

The clinical examinations were performed in the Otologic Center, Shandong Provincial ENT Hospital Affiliated to Shandong University. After physical and otoscopic examinations, pure tone audiometry (PTA) was performed. Other diseases or ototoxic medication history that may cause hearing loss were ruled out. The study was approved by the ethics committee of the Shandong Provincial ENT Hospital and written informed consents were obtained.

### 2.2 NGS of deafness gene

For mutation analysis, genomic DNA of the probands was extracted from peripheral blood using DNA extraction kit (Axygen) and subjected to a targeted NGS including 127 deafness‐related genes (see Table S1). Data were analysed in accordance with NGS standard process. Sequence alignment was performed by using BWA 0.6.2‐r126 software. UCSC hg19 Feb.2009 was used as reference genome. Mutation identification was performed by using GATK. dbSNP (snp137) as a reference. The pathogenicity of novel mutations was predicted by using 1000 genome database (phase I), HapMap database (combined data from phases II and III) and own databases as references. Guideline of American College of Medical Genetics and Genomics was used as the reference of data interpretation.^17^

### 2.3 Mutation detection by Sanger sequencing on genomic DNA

Sanger sequencing of *POU4F3* was performed using those primers: forward 5′‐CACCATCTGCAGGTTCGAGT‐3′ and reverse 5′‐CGAAATAGGCCTCGAGTGAAC‐3′ for c.704_705del mutation; forward 5′‐CACAGATCCATCCACACCAC‐3′ and reverse 5′‐CTTGCTGTTCTTCTCTCGGTAG‐3′ for c.593G>A mutation. Lasergene‐Seq Man software was used for data analysis. POU4F3 protein sequence (NP_002691.1) and gene (NM_002700) were used as references for sequence alignment.

### 2.4 Bioinformatics analysis

The mutations detected by targeted NGS was predicted for potential pathogenicity on Mutation Taster (http://www.mutationtaster.org/).^18^ The molecular structure of wild‐type (NP 002691.1, 338 amino acids) and mutant *POU4F3* was modelled using I‐TASSER (http://zhanglab.ccmb.med.umich.edu/). Protein visualization were performed using Swiss‐PdbViewer 4.1 software because of data from homology models.

### 2.5 Cell culture

The culture condition was as follow: MEM medium (Gibco) containing 10% FBS (Gibco), 5% CO_2_ at 37°C. Dr Federico Kalinec from University of California, Los Angeles friendly provided HEI OC1 auditory cells. The cells were maintained in DMEM medium with 10% FBS in 10% CO_2_ at 33°C.^19^

### 2.6 Plasmid construction

The plasmids loading *POU4F3* cDNA was obtained from Cusabio Biotech (Wuhan, China). The cDNA sequencing was confirmed by using Sanger sequencing (accessionnumber: BC112207). These *POU4F3* cDNA‐loaded plasmids were then used to amplify wild‐type POU4F3 by PCR reaction. The PCR Primers for *POU4F3* cDNA region were as follows: 5′‐ATGCAGGATCCATGATGGCCATGAACTCCAAGCAGCCTTTCG‐3′ and 5′‐ACGCAGAATTCGTGGACAGCCGAATACTTCA‐3′. The PCR products were digested by *BamH* I and *EcoR* I and cloned into pCMV‐Tag 2B plasmids (Agilent Technologies). To construct plasmids containing mutated *POU4F3*, the Quik Change site‐directed mutagenesis kit (Stratagene) was used to introduce the mutations (c.704_705del or c.593G>A) into the wild‐type vector.

### 2.7 Transient transfection and immunofluorescence analysis

HEI OC1 and HEK 293 cells were plated into 24 well plates. When cells were cultured to 80% confluence, the wild‐type or mutated *POU4F3* plasmids were transfected into both cell lines using Lipofectamine 3000 transfection reagent (Invitrogen). After transfection for 72 hours, cells were fixed in 4% paraformaldehyde for 15 minutes, permeabilized in PBS containing 0.3% Triton X‐100 for 10 minutes and then blocked in PBS containing 10% donkey serum for 1 hour at 37C in a humid atmosphere.

The cells were then incubated with primary anti‐FLAG antibody (Ca# F1804, Sigma, 1:800) for about 13 hours at 4°C. Sequentially, the secondary goat antimouse antibody (Sigma) and DAPI were incubated with cells with a dilution rate of 1:1000. Immunofluorescence imaging was performed by using a confocal microscope (TSC SPE, Leica).

### 2.8 Western blot

Total cell lysates extracted from the wild‐type or mutated *POU4F3* plasmids transfected HEK 293 cells were subjected to sodium dodecyl sulphate‐polyacrylamide gel electrophoresis and examined by Western blot with mouse monoclonal anti‐Flag antibodies (ZSGB‐Bio) and mouse monoclonal anti‐β‐actin antibodies (ZSGB‐Bio), and then followed by antimouse IgG conjugated horseradish peroxidase (ZSGB‐Bio). The signals were detected using the enhanced chemiluminescence system.

## 3 RESULTS

### 3.1 Clinical features

Two three‐generation Chinese families with ADNSHL were enrolled in this study. The pedigree and disease statements of all participants were shown in Figure 1A. Totally, there were seven members in F052* family and five members in F493* family suffered from similar hereditary progressive hearing loss, with 1 in F052* and 1 in F493* passed way at the time of investigation. As a result of patients' privacy and other special circumstances, we only got six members in F052* family and five members in F493* family to performed gene sequencing.

**FIGURE 2:**
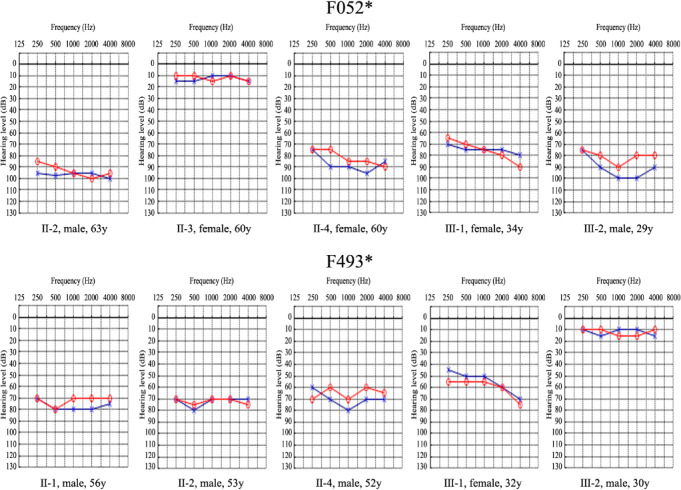
Audiograms of some members participating in this study in the two Chinese families. Blue crosses and red circles represent the air conduction hearing threshold levels of left and right ears, respectively. Asterisks indicate the families with POU4F3 mutations identified in this study. Gender and age are shown below the audiogram of each individual

According to the audiograms in Figure 2, all the affected individuals in two families, ageing from 29 years to 63 years, had severe hearing loss with a relatively ‘flat’ audiometric profile affecting all frequencies. Based on self‐description of patients with c.704_705del in F052* and c.593G>A in F493*, the hearing loss associated with these two mutations was typically post‐lingual, late onset and progressive (Table 1). For example, the proband III‐2 in F052* recalled that the onset age of bilateral NSHL was 10 and symptoms became worse after then.

**TABLE 1:** Summary of clinical data for members in hearing loss families

Patient	Gender	Age at test (y)	Age at onset	Use of aminoglycoside	PTA (dB) right ear	PTA (dB) left ear	Level of hearing impairment
F052* Ⅱ‐2	Male	63	20	No	96.5	95	Profound
F052* Ⅱ‐3	Female	60	—	No	12.5	12.5	Normal
F052* Ⅱ‐4	Female	60	30	No	90	83.75	Profound
F052* Ⅲ‐1	Female	34	15	No	78.75	76.25	Severe
F052* Ⅲ‐2	Male	29	10	No	95	82.5	Profound
F493* Ⅱ‐1	Male	56	17	No	78.75	72.5	Severe
F493* Ⅱ‐2	Male	53	20	No	72.5	72.5	Severe
F493* Ⅱ‐4	Male	52	12	No	72.5	63.75	Severe
F493* Ⅲ‐1	Female	32	17	No	61.25	57.5	Moderate
F493* Ⅲ‐2	Male	30	—	No	12.5	12.5	Normal

### 3.2 Identification of pathogenic *POU4F3* mutations in two Chinese ADNSHL families by targeted NGS

Here, we aimed to screen possible pathogenic mutations in probands of another two Chinese ADNSHL families (F052*‐III‐2 and F493*‐II‐2, Figure 1A) by using targeted NGS of 127 deafness‐related genes. As shown in Figure 1A, two novel mutations on *POU4F3*, c.704_705del (p.T235fs) and c.593G>A (p.R198H), were, respectively, identified as potential pathogenic variants in F052*‐III‐2 and F493*‐II‐2. These deleterious and pathogenic aspects of those two mutations are listed in Table 2.

**TABLE 2:** Characteristics of POU4F3 variant, analysis of predicted protein structure and disease‐causing effects

Gene	Exon	Domain	Variation				Polyphen‐2	Mutation Taster	SIFT	ExAC
			Nucleotide^a^	Amino acid^a^	Type	Status				
POU4F3	2	POU	c.593G>A	p.R198H	Missense	Heter	1	0.999848	0	Novel
POU4F3	2	POU	c.704_705del	p.235_235del	Frameshift	Heter	0.842	0.999824	0	Novel

Abbreviations: c, variation at cDNA level; ExAC, Exome Aggregation Consortium; Heter, heterozygote; p, variation at protein level; POU4F3, POU class 4 homeobox 3 (NM_002700).

^a^ All nucleotide and amino acid are abbreviated according to the International Union of Pure and Applied Chemistry (IUPAC).

Next, we used Sanger sequencing to confirm the two *POU4F3* variants in all the participating members. The results showed that c.704_705del and c.593G>A were the only *POU4F3* variants co‐separating with hear loss symptoms in F052* and F493*, respectively (Figure 1A,B). By searching online, we confirmed that c.704_705del and c.593G>A on *POU4F3* were not reported so far. Furthermore, we also used Sanger sequencing investigate these two mutations in 200 control individuals and none of them had identical mutations.

**FIGURE 3:**
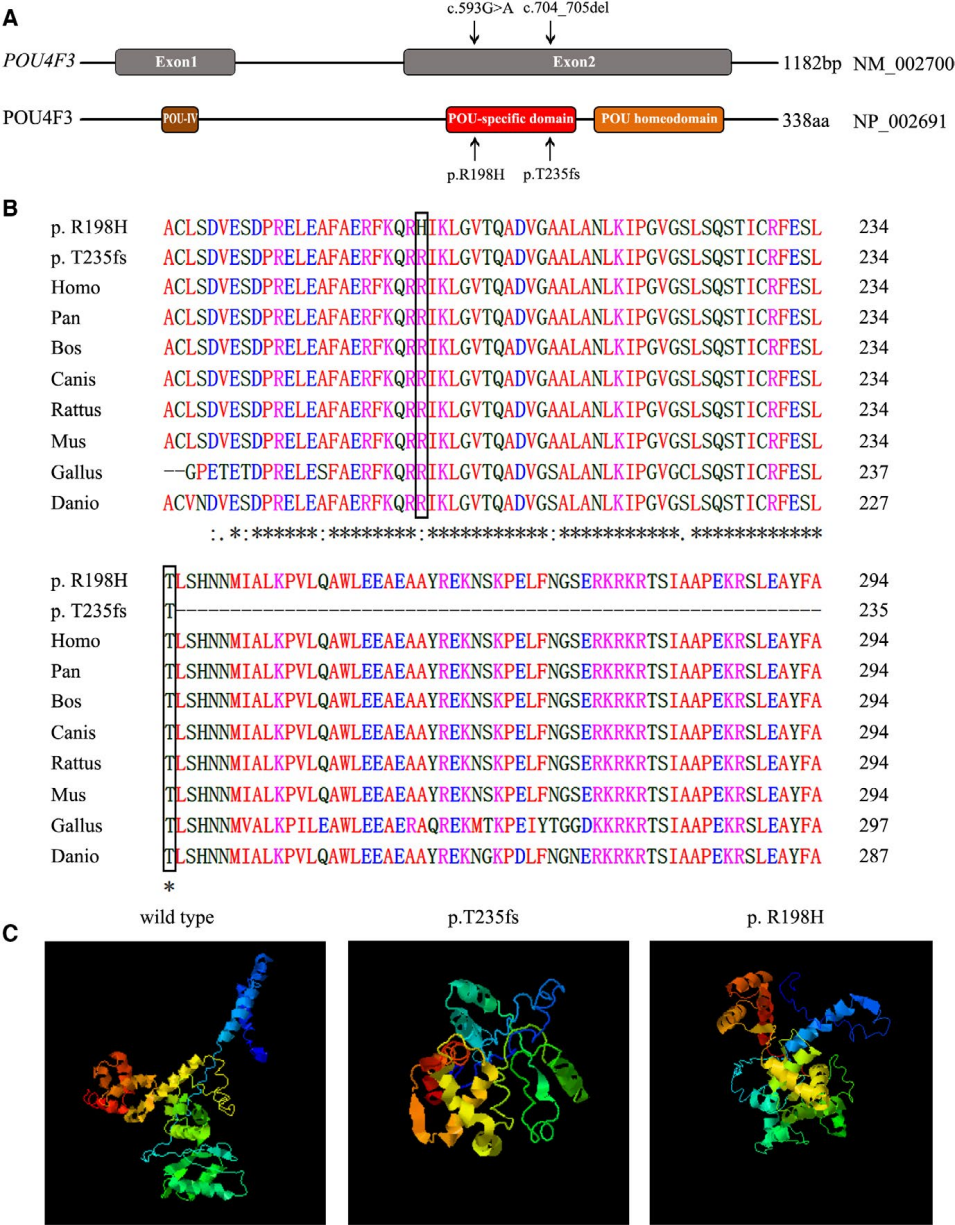
Genomic structure, conservation and 3D molecular model of POU4F3 mutations. A, Genomic structure of POU4F3 based on the open reading frame (NM_002700) containing two exons (grey rectangles). The positions of the c.704_705del (p.T235fs) and the c.593G>A (p.R198H) mutations are arrowed both at the gene (top) and the protein level (bottom). B, Protein alignment showing POU4F3 p.R198H occurred at evolutionarily conserved amino acids (in black box) across eight species. The mutation of c.704_705del (p.T235fs) caused a truncated protein. C, Three‐dimensional molecular models revealed the abnormal incomplete structure of mutant‐type protein

We analysed the effects of screened mutations of *POU4F3* on its protein structure. As shown in Figure 3A, both c.704_705del and c.593G>A occurred on exon 2 of *POU4F3*. The resulted amino acids variations p.T235fs and p.R198H were located on the evolutionarily conserved POU‐specific domain (Figure 3A).

The mutation of c.704_705del (p.T235fs) is a non‐sense mutation, which leads to a truncated protein. While, the missense mutation c.593G>A was predicted to lead to arginine to histidine amino acid substitution at codon 198. The mutation of c.704_705del (p.T235fs) caused a truncated protein. The alignment of POU4F3 from different species of the patients, *Homo*, *Pan*, *Bos*, *Canis*, *Rattus*, *Mus*, *Gallus* and *Danio* was shown in Figure 3B.

The three‐dimensional structures of wild‐type and mutant‐type *POU4F3* were simulated according to the crystal structure. As shown in Figure 3C, compared with the wild‐type structure, p.T235fs protein exhibited an abnormal truncated structure, resulted from the c.704_705del frameshift mutation. While, in p.R198H protein, the c.593G>A missense variant was predicted to perturb protein structure because of the substitution of arginine by histidine acid. In this respect, our prediction study revealed that these two novel mutations possibly lead to protein dysfunction.

### 3.3 Variations of p.T235fs and p.R198H alter the subcellular localization of POU4F3

Accumulated evidence demonstrated that *POU4F3* functions as an important transcription factor for the functions of hair cells.^13^, ^20^ So the subcellular localization of POU4F3 in nuclei is necessary for its function, because POU4F3 needs to combine to target DNA sequence. To investigate whether the pathogenicity of *POU4F3* mutations was mediated by the alteration of protein subcellular localization, we overexpressed wild‐type and mutant‐type *POU4F3* by using pCMV‐Tag2B plasmids in cell lines of HEK 293 and HEI OC1, an immortalized model of Corti‐derived epithelial cell line.

**FIGURE 4:**
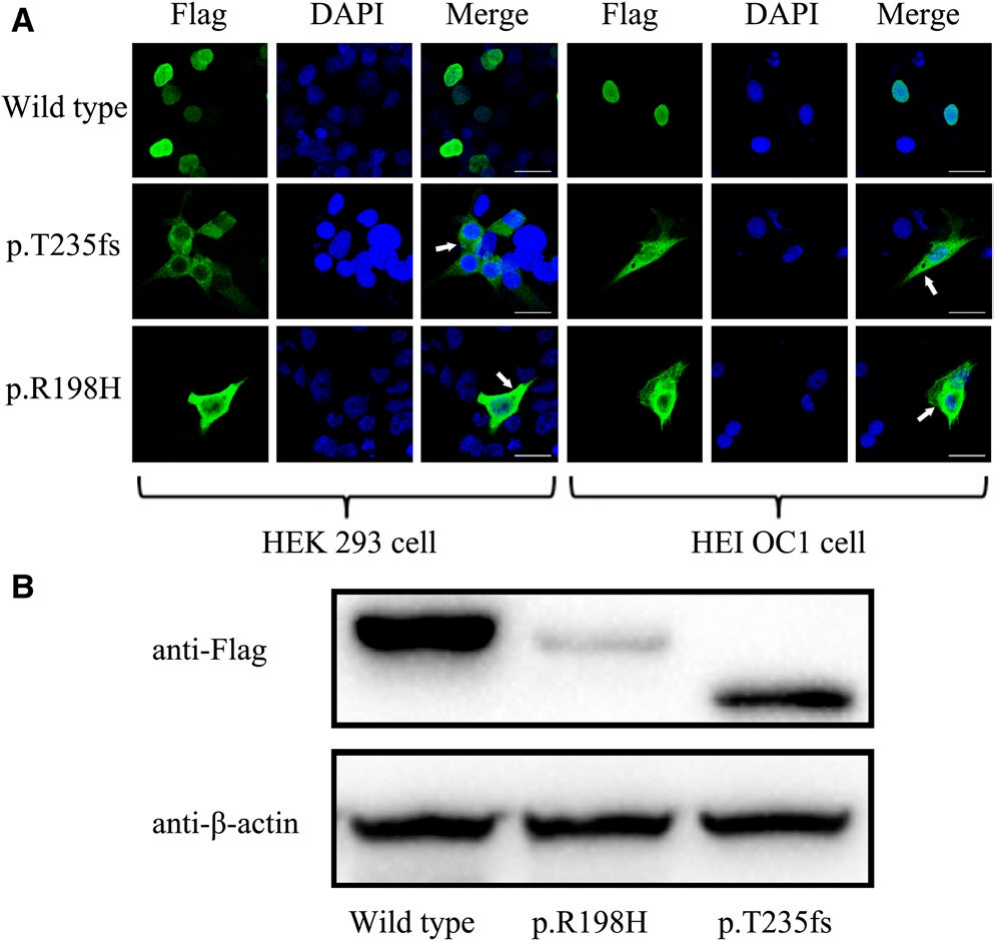
The POU4F3 c.704_705del and c.593G>A mutations altered the subcellular localization and protein expression of transcription factor POU4F3. A, Immunofluorescence staining was performed after transient transfection in HEK 293 cells and HEI OC1 cells. Images display DAPI in blue, Flag‐tagged protein in green, and merged pictures. Both in HEK 293 cells and HEI OC1 cells, mutant protein located mostly in cytoplasm while the wild‐type protein was exclusively located in nuclei. The white arrows indicate POU4F3 outside the nuclei. Scale bars = 30 µm. B, The expression of POU4F3 protein and its mutants in transfected HEK 293 cells were detected by immunoblotting using anti‐Flag and anti‐β‐actin (serving as an internal control) antibodies

As a FLAG‐tag was fused to the N‐terminal of POU4F3 protein, the subcellular localization of POU4F3 was detected by using anti‐FLAG immunofluorescence analysis. As shown in Figure 4A, wild‐type POU4F3 protein was exclusively located in the cell nuclei in both cell lines, while mutant‐type POU4F3 exhibited a strong fluorescence signal in cytoplasm and a much weaker in nuclei. These results suggested that variations of p.R198H and p.T235fs caused a retreat of POU4F3 from nuclei, which might explain the pathogenicity of screened mutations.

### 3.4 Mutations of p.T235fs and p.R198H affect POU4F3 protein expression

In order to detect the expression of mutant POU4F3 protein, the wild‐type POU4F3 plasmid and two mutant plasmids were transfected into HEK 293 cells, respectively. Western blot analysis of lysates abstracted from transfected HEK 293 cells was performed by immunoblotting using anti‐Flag and anti‐β‐actin antibodies. The result (Figure 4B) showed that the wild‐type and the p.R198H POU4F3 proteins were expressed as a single protein of the correct molecular weight, meaning that the staining observed in the immunocytochemical analysis is specifically derived from the tagged POU4F3 proteins. While a weak band of p.R198H POU4F3 protein was detected, indicating that the stability of p.R198H POU4F3 differed from that of the wild‐type protein. In addition, the p.T235fs POU4F3 protein was detected with a smaller molecular weight, implying this mutation result in a frameshift and premature termination of the POU4F3 protein.

## 4 DISCUSSION

POU4 family of transcription factors, with three members of POU4F1 ~ 3, are well‐known regulators in the development of sensory system. POU4F1 and POU4F2 are mainly expressed in retinal ganglion cells and involved in maintaining visual nervous system.^12^ In contrast, POU4F3 is uniquely expressed in the hair cells.^4^, ^5^ These three members all contain two common domains, POU homeodomain and POU‐specific domain, which were responsible for their DNA binding activity. *POU4F3* is involved in the proliferation and differentiation of hair cells by regulating the expression of neurotrophin, stress response proteins and so on.^8^, ^9^, ^10^, ^11^ In mice, targeted deletion of *POU4F3* led to the elimination of hair cells and severe hearing loss.^7^ Correspondingly, mutations of *POU4F3* also caused human late onset, progressive hearing loss (DFNA15) in multiple families.^21^, ^22^, ^23^, ^24^

Here, we identified c.704_705del and c.593G>A mutations of *POU4F3* as pathogenic causes of ADNSHL in F052* and F493* families, respectively. Both families manifested later onset, progressive hearing loss with an autosomal dominant pattern. The mutation c.704_705del caused a frameshift mutation, which altered the amino acid sequence after T235 and c.593G>A was a missense mutation causing p.R198H. Both mutations were located on the POU‐specific domain of POU4F3 and molecular model indicated that POU4F3 with either of them presented an abnormal structure. So mutations of c.704_705del and c.593G>A might disrupt the transcriptional activity of POT4F3 and further induced apoptosis of hair cells and hearing loss.

Since mutation of *POU4F3* was first discovered as a pathogenic cause for hearing loss in 1998,^12^ scientist and clinicians have identified 28 variants in *POU4F3* (Table 3) and a complete loss of *POU4F3* to be associated with ADNSHL. The affected individuals show a high degree of heterogeneity, including onset ages, disease levels and ethnicities. Epidemiological investigation have indicated that *POU4F3* mutations were one of the most common cause of ADNSHL in Japanese (15/602) and Chinese (8/13) families.^25^ In addition, as the severity of POU4F3 protein structural damage increases, patients' onset ages become earlier and disease progression slower.^21^ It is worth noting that most pathogenic mutations of *POU4F3* are located on POU‐domains, responsible for DNA‐binding function of POU4F3.

**TABLE 3:** POU4F3 gene mutations found in patients with DFNB15

Nucleotide change	Protein change	Exon	Domain	Ethnicity	Age of HL diagnosis	Severity of HL	Progression of HL	Audiometric configuration	Reference
Whole deletion of POU4F3				Brazil	11‐13 y	Moderate to profound	Yes	Flat and HF	29
c.74dupA	p.His25fs*18	1		Japan	20's	Profound	Yes	HF	21
c.120+1G>T		1		China	0‐40 y	Moderate to profound	Yes	Flat	31
c.191A>T	p.Asp64Val	2		Japan	10‐30 y	Moderate to profound	Yes	HF	21
c.337C>T	p.Gln113Ter	2		China	14‐40 y	Moderate to severe	Yes	Flat and HF	24
c.367delA	p.Ile123fs*3	2		Japan	40's	Moderate	Yes	MF	21
c.427C>	p.Gln143Ter	2		Japan	3 y	Moderate	NA	MF	21
c.491C>G	p.Pro164Arg	2	POU	China	NA	Mild to profound	NA	Flat and HF	14
c.574G>T	p.Glu192Ter	2	POU	Japan	17‐30's	Moderate	Yes	MF	21
c.581T>A	p.Phe194Tyr	2	POU	Japan	10‐20 y	Moderate	Yes	MF and HF	21
c.593G>A	p.Arg198His	2	POU	China	28‐56 y	Severe	Yes	Flat	This study
c.602T>C	p.Leu201Pro	2	POU	China	10's	Mild to profound	Yes	MF	25
c.602delT	p.Leu201fs	2	POU	China	16‐30 y	Mild to profound	Yes	HF	32
c.603_604delGG	p.Val203fs	2	POU	China	NA	NA	NA	NA	33
c.662_675del14	p.Gly221fs	2	POU	Korea	20 y	Severe	NA	HF	22
c.665C>T	p.Ser222Leu	2	POU	Japan	6 y	Moderate	Yes	HF	21
c.668T>C	p.Leu223Pro	2	POU	Netherland	4‐44 y	Mild to severe	Yes	Flat, MF and HF	13
c.680delC	p.Thr227fs*13	2	POU	Japan	Infant	Moderate to severe	Yes	MF and HF	21
c.694G>A	p.Glu232Lys	2	POU	Korea	20's	Moderate to severe	NA	HF	34
c.704_705del	p.Thr235fs	2	POU	China	27‐63 y	Profound	Yes	Flat	This study
c.718A>T	p.Asn240Tyr	2	POU	Japan	6 y	Moderate	Yes	MF	21
c.841A>G	p.Ile281Val	2	POU Homeobox	Japan	50‐54 y	Moderate	Yes	HF	21
c.865C>T	p.Leu289Phe	2	POU Homeobox	Netherland	13‐20 y	Mild to profound	Yes	Flat, MF and HF	13
c.884_891del8	p.Ile295fs	2	POU Homeobox	Israel	18‐30 y	Moderate to severe	Yes	HF	12
c.896C>T	p.Pro299Leu	2	POU Homeobox	Japan	26‐27 y	Moderate to profound	Yes	Flat, MF and HF	21
c.932T>C	p.Leu311Pro	2	POU Homeobox	China	10‐20 y	Moderate to profound	Yes	HF	31
c.976A>T	p.Arg326Ter	2	POU Homeobox	Japan	Childhood	Moderate	Yes	HF	21
c.977G>A	p.Arg326Lys	2	POU Homeobox	Korea	10‐50's	Mild to moderate	Yes	Flat and HF	15
c.982A>G	p.Lys328Glu	2	POU Homeobox	Taiwan	10's	Profound	Yes	HF	23
c.1007delC	p.Ala336fs	2	POU Homeobox	Japan	0 y	Moderate to severe	Yes	NA	30

Previous studies have demonstrated mutations that disrupted the nuclear localization sequence (NLS) resulted a defected trafficking of POU4F3 into cell nucleus, where it exerted transcriptional activity to regulate gene expression.^13^, ^24^ POU4F3 contained two NLSs, including a monopartite (amino acids 274‐278) and a bipartite (amino acids 314‐331). Yin‐Hung Lin et al reported that a missense mutation of p.Lys328Glu in bipartite NLS of POU4F3 could compromise transportation of POU4F3 from the cytoplasm to the nucleus.^23^ Previously, we also found that c.337C>T (p. Gln113∗) resulted into a truncated protein lacking the bipartite NLS and caused location of POU4F3 in cytoplasm.^24^ In this study, we investigated the effects of diagnosed mutations of POU4F3 on its subcellular localization. In vitro experiments indicated that POU4F3 with either c.704_705del (p.T235fs) or c.593G>A (p.R198H) was mostly located in cytoplasm, while wild‐type protein were immobilized in the nucleus. So we speculated that these two mutations (a frameshift and a missense mutation) disrupted the sequence or structure of POU4F3 NLS domain.

Although the mechanism of deafness caused by *POU4F3* mutations is not well studied, the results of several studies point to haploinsufficiency. Our study strongly indicated that both missense mutations p.R198H as well as the frameshift mutation p.T235fs affect proper functioning of the POU4F3 protein. In terms of molecular biology, dominant inheritance involved three mechanisms: haploinsufficiency, function‐gaining and dominant‐negative effect.^26^ As a dominant‐negative effect have been ruled out, haploinsufficiency is the most likely mechanism so far.^13^, ^27^ Although mice with Brn3c^−/−^ behaved healthily, several studies in humans supported the haploinsufficiency mode.^28^ Freitas, E. L. et al reported that a complete deletion of *POU4F3* caused ADNSHL.^29^ Mutai, H. et al reported a frameshift variant of POU4F3 in a Japanese family, which would produce an extended mRNA because of the absence of in‐frame stop codon.^30^ The overlong mRNAs might be degraded in cells. In other words, these two mutations make *POU4F3* a haplotype, which could only be explained by haploinsufficiency. Furthermore, the altered protein localization of POU4F3 in this study also supports haploinsufficiency mechanism.

In summary, by using a targeted NGS‐base genetic test, we identified two novel variants of *POU4F3*, c.704_705del (p.T235fs) and c.593G>A (p.R198H), in two Chinese families with ADNSHL, respectively. Function analysis suggested that these two mutations caused an abnormal, incomplete protein structure and impaired nuclear localization of POU4F3. This study provided new knowledge of *POU4F3* mutation spectrum associated with hearing loss and helps to identify the pathogenic mechanism of *POU4F*.

## CONFLICT OF INTEREST

The authors declare that they have no conflict of interest.

## AUTHOR CONTRIBUTIONS

XB and LX were in charge of the idea, project design and concept of the study; XB performed the in vivo experiments; FZ, YX and YJ performed DNA extraction, PCR amplification, sequencing and data analysis; HW and LX recruited the clinical patients and were in charge of the clinical assessments; XB wrote the manuscript; QZ and LX revised the manuscript. All authors read and approved the manuscript.

## Supporting information

Table S1

## Data Availability

The data that support the findings of this study are available from the corresponding author upon reasonable request.
