# Supplementary material for: Identification of two novel mutations in POU4F3 gene associated with autosomal dominant hearing loss in Chinese families
Source: J Cell Mol Med. 2020 May 11;24(12):6978–87. doi: 10.1111/jcmm.15359 (PMC7299729; doi:10.1111/jcmm.15359)
Supplement: Supplementary file 1 — Table S1 [file JCMM-24-6978-s001.docx]

Supplementary Table 1. Summary of the 127 targeted deafness genes.

| Gene | NS/S | Inheritance | mRNA | Exons |
| --- | --- | --- | --- | --- |
| ACTG1 | NS | AD | NM_001199954.1 | 6 |
| ALMS1 | S | AR | NM_015120.4 | 23 |
| ATP2B2 | NS | AR | NM_001001331.2 | 23 |
| BSND | NS/S | AR | NM_057176.2 | 4 |
| CACNA1D | NS/S | AR | NM_000720.3 | 49 |
| CCDC50 | NS | AD | NM_178335.2 | 12 |
| CDH23 | NS/S | AR | NM_022124.5, | 70 |
| CEACAM16 | NS | AD | NM_001039213.3 | 7 |
| CHD7 | S | AD | NM_017780.3 | 38 |
| CLDN14 | NS | AR | NM_144492.2 | 3 |
| CLRN1 | NS/S | AR | NM_174878.2 | 3 |
| COCH | NS | AD | NM_001135058.1 | 11 |
| COL11A1 | S | AR/AD | NM_001854.3 | 67 |
| COL2A1 | S | AD | NM_033150.2 | 53 |
| COL4A3 | S | AR | NM_000091.4 | 52 |
| COL4A4 | S | AR | NM_000092.4 | 48 |
| COL4A5 | S | X-linked | NM_000495.4 | 51 |
| COL9A1 | S | AR | NM_001851.4 | 38 |
| COL9A2 | S | AR | NM_001852.3 | 32 |
| CRYM | NS | AD | NM_001888.4 | 10 |
| DFNA5 | NS | AD | NM_004403.2 | 10 |
| DFNB31 | NS/S | AR | NM_015404.3 | 12 |
| DFNB59/PJVK | NS | AR | NM_001042702.3 | 7 |
| DIABLO | NS | AD | NM_019887.5 | 7 |
| DIAPH1 | NS | AD | NM_005219.4 | 28 |
| DIAPH3 | NS | AD | NM_001258366.1 | 27 |
| DLX5 | S | AD | NM_005221.5 | 3 |
| DSPP | NS | AD | NM_014208.3 | 5 |
| EDN3 | S | AD | NM_207032.2 | 5 |
| EDNRB | S | AD | NM_000115.3 | 8 |

| ESPN | NS | AR/AD | NM_031475.2 | 13 |
| --- | --- | --- | --- | --- |
| ESRRB | NS | AR | NM_004452.3 | 11 |
| EYA1 | S | AD | NM_000503.5 | 18 |
| EYA4 | NS | AD | NM_004100.4 | 20 |
| FGF3 | S | AR | NM_005247.2 | 3 |
| FGFR1 | S | AD | NM_023110.2 | 18 |
| FGFR2 | S | AD | NM_000141.4 | 18 |
| FGFR3 | S | AD | NM_000142.4 | 18 |
| FOXI1 | NS/S | AR/AD | NM_012188.4 | 2 |
| GATA3 | S | AD | NM_001002295.1 | 6 |
| GIPC3 | NS | AR | NM_133261.2 | 6 |
| GJA1 | NS | AR | NM_000165.4 | 2 |
| GJB2 | NS | AR/AD | NM_004004.5 | 2 |
| GJB3 | NS | AR/AD | NM_024009.2 | 2 |
| GJB6 | NS | AR/AD | NM_001110219.2 | 5 |
| GLI3 | S | AD | NM_000168.5 | 15 |
| GPR98 | NS/S | AR | NM_032119.3 | 90 |
| GPSM2 | NS | AR | NM_013296.4 | 15 |
| GRHL2 | NS | AD | NM_024915.3 | 16 |
| GRXCR1 | NS | AR | NM_001080476.2 | 4 |
| HGF | NS | AR | NM_000601.4 | 18 |
| HOXA1 | S | AR | NM_005522.4 | 2 |
| HOXA2 | S | AR | NM_006735.3 | 2 |
| IGF1 | S | AR | NM_001111283.1 | 5 |
| ILDR1 | NS | AR | NM_001199799.1 | 8 |
| KCNE1 | NS/S | AR | NM_000219.5 | 4 |
| KCNJ10 | NS/S | AR | NM_002241.4 | 2 |
| KCNQ1 | NS/S | AR | NM_000218.2 | 16 |
| KCNQ4 | NS | AD | NM_004700.3 | 14 |
| LHFPL5 | NS | AR | NM_182548.3 | 4 |
| LOXHD1 | NS | AR | NM_144612.6 | 40 |
| LRP2 | S | AR | NM_004525.2 | 79 |
| LRTOMT | NS | AR | NM_145309.5 | 6 |

| MARVELD2 | NS | AR | NM_001038603.2 | 7 |
| --- | --- | --- | --- | --- |
| MIR96 | NS | AD | NR_029512.1 |  |
| MITF | S | AD | NM_198159.2 | 10 |
| MSRB3 | NS | AR | NM_198080.3 | 6 |
| MT-RNR1 | NS | MT | — |  |
| MT-TE | S | MT | — |  |
| MT-TK | S | MT | — |  |
| MT-TL1 | S | MT | — |  |
| MT-TS1 | NS | MT | — |  |
| MYH14 | NS | AD | NM_001077186.1 | 42 |
| MYH9 | NS | AD | NM_002473.5 | 41 |
| MYO15A | NS | AR | NM_016239.3 | 66 |
| MYO1A | NS | AD | NM_001256041.1 | 29 |
| MYO3A | NS | AR | NM_017433.4 | 35 |
| MYO6 | NS | AR/AD | NM_004999.3 | 35 |
| MYO7A | NS/S | AR/AD | NM_000260.3 | 49 |
| NDP | S | X-linked | NM_000266.3 | 3 |
| OPA1 | S | AD | NM_015560.2 | 29 |
| OTOA | NS | AR | NM_144672.3 | 28 |
| OTOF | NS | AR | NM_194248.2 | 47 |
| OTOG | NS | AR | NM_001277269.1 | 55 |
| PAX2 | S | AD | NM_003987.3 | 11 |
| PAX3 | S | AD | NM_181457.3 | 8 |
| PCDH15 | NS/S | AR | NM_001142765.1 | 32 |
| PDSS1 | S | AR | NM_014317.3 | 12 |
| PDZD7 | NS/S | AR | NM_001195263.1 | 17 |
| PHEX | S | X-linked | NM_000444.5 | 22 |
| POU3F4 | NS | X-linked | NM_000307.4 | 1 |
| POU4F3 | NS | AD | NM_002700.2 | 2 |
| PRPS1 | NS | X-linked | NM_002764.3 | 7 |
| PRRX1 | S | AR | NM_022716.3 | 4 |
| PTPRQ | NS | AR | NM_001145026.1 | 45 |
| RDX | NS | AR | NM_001260492.1 | 16 |

| SEMA3E | S | AR | NM_012431.2 | 17 |
| --- | --- | --- | --- | --- |
| SERAC1 | S | AR | NM_032861.3 | 17 |
| SERPINB6 | NS | AR | NM_001297699.1 | 7 |
| SIX1 | NS/S | AD | NM_005982.3 | 2 |
| SIX5 | S | AD | NM_175875.4 | 3 |
| SLC17A8 | NS | AD | NM_139319.2 | 12 |
| SLC19A2 | S | AR | NM_006996.2 | 6 |
| SLC26A4 | NS/S | AR | NM_000441.1 | 21 |
| SLC26A5 | NS | AR | NM_198999.2 | 20 |
| SLC4A11 | S | AR | NM_001174090.1 | 20 |
| SMAD4 | S | AD | NM_005359.5 | 12 |
| SMPX | NS | X-linked | NM_014332.2 | 5 |
| SNAI2 | S | AD | NM_003068.4 | 3 |
| SOBP | S | AR | NM_018013.3 | 7 |
| SOX10 | S | AD | NM_006941.3 | 4 |
| SOX9 | S | AD | NM_000346.3 | 3 |
| STRC | NS | AR | NM_153700.2 | 29 |
| TCOF1 | S | AD | NM_001135244.1 | 26 |
| TECTA | NS | AR/AD | NM_005422.2 | 23 |
| TIMM8A | NS/S | X-linked | NM_004085.3 | 2 |
| TJP2 | NS | AD | NM_004817.3 | 23 |
| TMC1 | NS | X-linked | NM_138691.2 | 24 |
| TMIE | NS | AR | NM_147196.2 | 4 |
| TMPRSS3 | NS | AR | NM_024022.2 | 13 |
| TNFRSF11B | S | AD | NM_002546.3 | 5 |
| TPRN | NS | AR | NM_001128228.2 | 4 |
| TRIOBP | NS | AR | NM_138632.2 | 8 |
| USH1C | NS/S | AR | NM_005709.3 | 21 |
| USH1G | NS/S | AR | NM_173477.4 | 3 |
| USH2A | NS/S | AR | NM_206933.2 | 72 |
| WFS1 | NS/S | AR/AD | NM_006005.3 | 8 |

NS: Non-Syndromic; S: Syndromic; AR: Autosomal Recessive; AD: Autosomal Dominant; MT: Mitochondrial Inheritance
